# Involvement of information professionals in patient- and family-centered care initiatives: a scoping review

**DOI:** 10.5195/jmla.2019.652

**Published:** 2019-07-01

**Authors:** Antonio P. DeRosa, Becky Baltich Nelson, Diana Delgado, Keith C. Mages, Lily Martin, Judy C. Stribling

**Affiliations:** Oncology Consumer Health Librarian, Samuel J. Wood Library & C.V. Starr Biomedical Information Center, Weill Cornell Medical College, New York, NY, apd2004@med.cornell.edu; Clinical and Systems Librarian, Samuel J. Wood Library & C.V. Starr Biomedical Information Center, Weill Cornell Medical College, New York, NY, blb2008@med.cornell.edu; Associate Director, Information, Education and Clinical Services, Samuel J. Wood Library & C.V. Starr Biomedical Information Center, Weill Cornell Medical College, New York, NY, did2005@med.cornell.edu; Clinical Medical Librarian, Samuel J. Wood Library & C.V. Starr Biomedical Information Center, Weill Cornell Medical College, New York, NY, kcm2001@med.cornell.edu; Health Sciences Librarian, Daniel Carroll Payson Medical Library, North Shore University Hospital, Manhasset, NY, lmartin16@northwell.edu; Assistant Director, Clinical Services, Samuel J. Wood Library & C.V. Starr Biomedical Information Center, Weill Cornell Medical College, New York, NY, jcs2002@med.cornell.edu

## Abstract

**Objective:**

The goal of this scoping review was to collect data on patient- and family-centered care (PFCC) programs and initiatives that have included the direct involvement of librarians and information professionals to determine how librarians are involved in PFCC and highlight opportunities for librarians to support PFCC programs.

**Methods:**

Systematic literature searches were conducted in seven scholarly databases in the information, medical, and social sciences. Studies were included if they (1) described initiatives presented explicitly as PFCC programs and (2) involved an information professional or librarian in the PFCC initiative or program. Based on the definition of PFCC provided by the Institute for Patient- and Family-Centered Care, the authors developed a custom code sheet to organize data elements into PFCC categories or initiatives and outcomes. Other extracted data elements included how the information professional became involved in the program and a narrative description of the initiatives or programs.

**Results:**

All included studies (n=12) identified patient education or information-sharing as an integral component of their PFCC initiatives. Librarians were noted to contribute to shared decision-making through direct patient consultation, provision of health literacy education, and information delivery to both provider and patient with the goal of fostering collaborative communication.

**Conclusions:**

The synthesis of available evidence to date suggests that librarians and information professionals should focus on patient education and information-sharing to support both patients or caregivers and clinical staff. The burgeoning efforts in participatory care and inclusion of patients in the decision-making process pose a unique opportunity for librarians and information professionals to offer more personalized information services.

## INTRODUCTION

The adoption of patient- and family-centered care (PFCC) is increasing across the national health care landscape [1]. This approach emphasizes patients and their families as critical partners throughout the entirety of the health care process. In addition to its focus on the patient-physician relationship, PFCC extends to all who interact with patients or impact their care, including librarians and other information professionals.

Launched in 1992, the Institute for Patient- and Family-Centered Care (IPFCC) is a nonprofit organization that works to change the provision of health care services by integrating PFCC into each facet of the health care system. The IPFCC provides national and international leadership for advancing the practice of PFCC by promoting collaborative partnerships among patients, families, and health care professionals. They provide information and resources to any interested group—from policy makers to direct service providers—and advance PFCC through “education, consultation, and technical assistance; materials development and information dissemination; research; and strategic partnerships.” In 2014, the IPFCC launched the Better Together: Partnering with Families initiative, which challenged hospitals’ restrictive visiting rights. This initiative has since widened to include partnerships with the American Society for Healthcare Risk Management, American Association of Critical Care Nurses, Ronald McDonald House Charities, National Partnership for Women and Families, Canadian Foundation for Healthcare Improvement, New Yorkers for Patient and Family Empowerment, Health in Aging Foundation, and Daisy Foundation [2].

The IPFCC identifies four core concepts of PFCC: dignity and respect, information-sharing, participation, and collaboration. These core concepts recognize that health care improves when patients and their families have their perspectives and beliefs incorporated into care, when they receive accurate and level-appropriate information, and when they are encouraged to participate in decision-making for their own care and to collaborate beyond their own care to improve policies, programs, facilities, research, and education.

The goal of this scoping review was to collect data on PFCC programs and initiatives that included the direct participation of a consumer health librarian or other information professional. As PFCC becomes more widely adopted across the health care system, it will be useful for librarians to know the various ways in which they can participate in these activities and provide support to patients, families, and health care professionals.

## METHODS

The protocol for this scoping review research was developed following Institute of Medicine standards and was registered with the International Prospective Register of Systematic Reviews (PROSPERO) through the National Institute for Health Research and University of York Centre for Reviews and Dissemination (#CRD42018093074).

On July 19–20, 2018, the authors conducted comprehensive literature searches in seven scholarly or scientific databases for English-language papers with no specified limits on dates of publication. The databases searched were: (1) MEDLINE (via Ovid); (2) Embase (via Ovid); (3) the Cochrane Library; (4) Web of Science; (5) CINAHL (via EBSCO); (6) Library Literature & Information Science Index (via EBSCO); and (7) Library, Information Science, & Technology (LISTA) (via EBSCO). Controlled vocabularies and keywords were used in search strategies for MEDLINE, Embase, the Cochrane Library, CINAHL, Library Literature & Information Science Index, and LISTA. Web of Science does not employ controlled vocabularies, so it was searched using only keywords.

The search strategy had two major components that were linked together with the Boolean AND operator: (1) terms related to information professionals (e.g., librarian, informationist, information specialist, information scientist) and (2) terms related to PFCC (e.g., patient centred/centered, patient navigation, family centred/centered, patient participation). To investigate the grey literature perspective of this research topic, we conducted comprehensive searches in Embase and Web of Science to include all publication types such as conference proceedings, research and other reports, and theses or dissertations. The following grey literature sources were also searched: (1) New York Academy of Medicine’s Grey Literature Report, (2) European Association for Grey Literature Exploitation’s OpenGrey resource, and (3) National Library of Medicine’s Health Services Research Projects in Progress (HRSProj) resource. A complete list of Medical Subject Headings (MeSH) terms and keywords used for the MEDLINE search strategy are provided in supplemental Appendix A.

All search results were imported into the systematic review support tool Covidence for reference management, duplicate title reduction, and screening. Independent screening of all references was undertaken in two phases: title/abstract and full-text screening. At least two reviewers evaluated each reference, with a third reviewer acting as tie-breaker when needed. The criteria for inclusion of studies contained two elements: (1) description and detail of patient-centered initiatives or projects explicitly presented as PFCC programs and (2) information professional or librarian involvement in the PFCC initiatives or programs. All research designs and publication types were eligible for inclusion.

The data extraction was conducted using a custom-developed code sheet (supplemental Appendix B). We extracted the following data elements from each study for qualitative analysis: (1) author, (2) year, (3) study design, (4) population, (5) PFCC category, (6) institution type, (7) librarian or information professional initiatives, (8) how librarians or information professionals initially became involved, and (9) PFCC outcome. Study design, population, and institution type have discreet response choices, as did PFCC category and outcome based on the IPFCC’s definition of PFCC [1], although we provided the option to write in other relevant PFCC categories or outcomes. Librarians’ or information professionals’ initiatives and involvement in PFCC programs were free-text fields where we could describe each initiative in detail.

## RESULTS

### Summary of included studies

**Figure 1:**
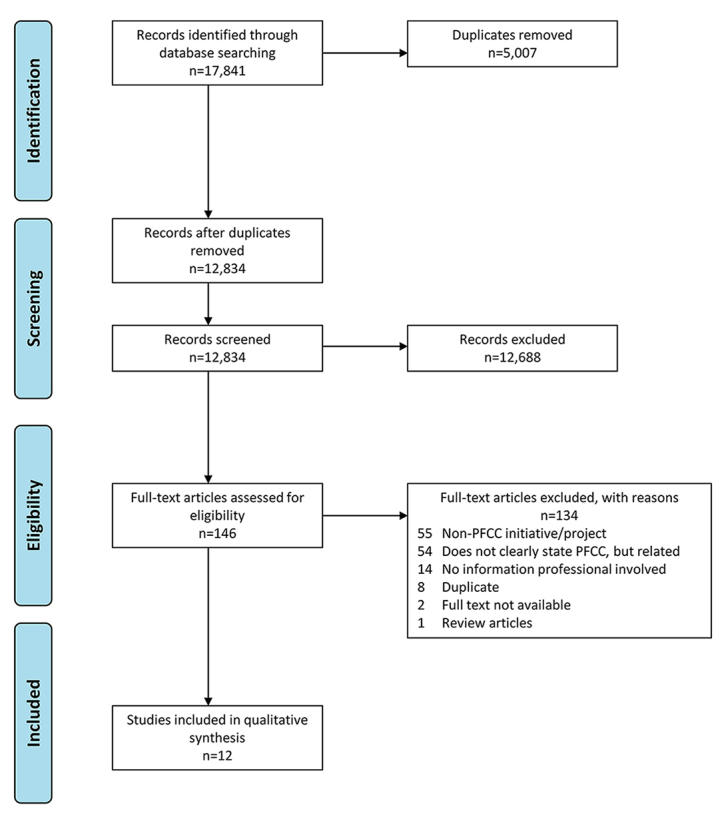
PRISMA flow diagram From Moher D, Liberati A, Tetzlaff J, Altman DG [15].

After extensive review of the literature (Figure 1), twelve studies met our criteria for inclusion [3–14], the details of which are provided in supplemental Appendix C. All of these studies were case reports, which was expected, based on the topic of the scoping review. The population described in each study was mixed, consisting of a combination of patients, families, caregivers, health professionals, and the public or surrounding community. All PFCC programs described in these studies took place in a hospital or academic medical center or university setting. The included studies were published between the years of 1997–2017.

PFCC category and outcomes characteristics were tallied across included studies. Regarding PFCC category, all studies described information-sharing or patient education programs, with participatory care or decision-making and education of health professional programs also frequently described (Table 1). Regarding PFCC outcome, improving patient and family experience was described in all studies, with other outcomes described less frequently (Table 2). Three studies described empowerment as an “other” outcome. Empowerment is described in these studies through patient or caregiver narrative feedback as to how information that was provided helped them to feel more confident in communicating with their care teams and asking questions.

**Table 1:** Number of included studies describing different patient- and family-centered care (PFCC) categories

Patient- and family-centered care (PFCC) category	n
Information-sharing/patient education	12
Participatory care/decision-making	8
Education of health professionals	6
Safety initiatives	1
Facility design	1
Policy development	1
Cultural and spiritual competencies	1
Quality/service improvement	0
Research	0

**Table 2:** Number of included studies describing different PFCC outcomes

PFCC outcome	n
Improved patient/family experience	12
Better health outcomes	4
Better clinician/staff satisfaction	4
Other* (empowerment)	3
Wiser allocation of resources	1

* Other=write-in category.

All included studies described how librarians or information professionals initially got involved in the PFCC programs and initiatives, which can be mainly categorized as invitations to participate or to perform committee work (n=6) or librarian- or information professional–initiated (n=6). The details of librarian or information professionals’ involvement in PFCC initiatives are described in the following section.

### Overviews of included studies

Anglin, a family health librarian at the Hassenfeld Children’s Center for Cancer and Blood Disorders at New York University (NYU) Langone Health, articulated the consumer health information services that were available at her outpatient hematology/oncology clinic. Librarian involvement in activities included health literacy evaluation and education, school readiness, patient psychosocial needs, and support, as well as targeted research support for both patients and clinicians. The librarian was described as a key member of the integrative care team. PFCC initiatives were many and included “education of health professionals,” “information-sharing/patient education,” and “participatory care/decision-making.” Unique to this article were discussions of librarian activities in PFCC “safety initiatives,” “facility design,” “policy development,” and “cultural and spiritual competencies.” The PFCC outcomes of “better health outcomes,” “improved patient/family experience,” and “better clinician/staff satisfaction” were reported, as evidenced by direct observation, individualized educational approaches, acquisition of culturally diverse library collections, and anecdotal conversations with patrons [3].

Babish from Moses Taylor Hospital in Scranton, Pennsylvania, reported on the PFCC initiatives she observed while visiting the Planetree Health Resource Center–affiliated Griffin Hospital in Derby, Connecticut. At this institution, Babish noted librarian involvement with health literacy initiatives as well as librarian-facilitated one-on-one sessions that were focused on improving patient knowledge and empowerment, activities that were consistent with the PFCC initiatives of “information-sharing/patient education” and “participatory care/decision-making.” These initiatives contributed to the PFCC outcome of “improved patient/family experience,” which was evidenced by the author’s direct observations [4].

Calabretta et al. highlighted PFCC programs at Cooper University Hospital in Camden, New Jersey. Programs with librarian involvement included consulting with patients, family members, or staff to provide targeted health information and promote patient-centered services to employees via open houses, benefit fairs, and direct communication with nursing staff. Identified PFCC initiatives included “education of health professionals” and “information-sharing/patient education.” PFCC outcomes consisted of “improved patient/family experience,” “better clinician/staff satisfaction,” and “wiser allocation of resources,” which were documented via user sign-in log data, staff referral numbers, and direct feedback from patients, family, or staff [6].

The editors of *Healthcare Demand and Disease Management* reported on the establishment and successes of the Planetree Health Resource Center of Mid-Columbia Medical Center in The Dalles, Oregon. Following the patient-as-partner model of Planetree health facilities, many programs of this resource center touched upon the PFCC initiatives of “education of health professionals” and “information-sharing/patient education.” Programs of note included the delivery of targeted health information materials to patients, including patient information on physician-assisted suicide. Of relevance to both patients and clinicians was the curation of resource guides on local, regional, and national support groups that were organized by specific diseases and/or diagnoses. The PFCC outcome of note was “improved patient/family experience,” based on user feedback and an increase in service requests [9].

Tarby and Hogan of Crouse Hospital in Syracuse, New York, documented collaborative patient education efforts that included the development of streamlined patient- and clinician-initiated information requests as well as participation in both direct education and the development of hospital-wide standard patient information materials. These activities coincided with the PFCC initiatives of “education of health professionals,” “information-sharing/patient education,” and “participatory care/decision-making.” Evaluation of these initiatives contributed to the PFCC outcomes of “better health outcomes” and “improved patient/family experiences,” which were identified through direct staff and patient feedback (garnered during the course of collaborative efforts) showing high patient satisfaction scores among units that required the most patient teaching and utilized the integrated collaborative information service model [12].

Truccolo, head librarian at the Scientific and Patient Library of the Comprehensive Cancer Center in Italy, highlighted several activities coinciding with the PFCC initiatives of “education of health professionals” and “information-sharing/patient education.” As coordinator of patient educational classes, Truccolo routinely surveyed and collected topic suggestions from patients to select health care professionals who could best educate patients on those topics. Furthermore, Truccolo harnessed her patient experiences to contribute to the development and evaluation of institutional patient education materials. Finally, she served as a coordinator and facilitator of institutional narrative medicine initiatives, such as an annual “artistic-literary competition” that provided a platform for patient expression. The most pertinent PFCC outcome was identified as “improved patient/family experience,” based upon author-provided observational evidence [13].

Davis discussed a collaboration between the consumer health librarian and the volunteer department at Sharp Memorial Hospital in San Diego, California, that involved the creation of a Health Information Ambassador Program. The goals of this program corresponded to the PFCC initiatives “information-sharing/patient education” and “participatory care/decision-making.” As a result of work done on the hospital’s Patient and Family Centered Care Team, volunteers were trained to round on hospital floors and pick up information requests from patients, families, and health professionals. These requests were forwarded to librarians who created customized information packets for patients. As a result of the program, Davis reported that the volunteers and librarians received a variety of information requests in several different languages. PFCC outcomes from this intervention included “improved patient/family experience” and “other (empowerment),” as evidenced by observations from volunteers and direct feedback from patients and families [7].

Similarly, at the Biomedical Library at Vanderbilt University Medical Center, Williams et al. described how patients were provided with consumer-friendly information via the Patient Informatics Consult Service (PICS). This service aligned with the PFCC initiatives of “information-sharing/patient education” and “participatory care/decision-making,” as it provided patients and their families with the information that they needed to become informed participants in their health care. In this initiative, specialized “information prescription” packets were filled out by clinicians and forwarded to PICS librarians [14]. A thorough description of information prescriptions can be found in McKnight’s excellent history of physician-ordered reading for patients [16]. The librarians at Vanderbilt, in turn, create tailored information packets that took into account a patient’s literacy level and information needs. The report was given to both physician and patient to ease doctor-patient communication and create a dialogue. The PFCC outcomes of this program included “better health outcomes,” “improved patient/family experience,” “better clinician/staff satisfaction,” and “other (empowerment),” as indicated by positive anecdotal feedback [14].

At the Komansky Center for Children’s Health pediatric floors of New York-Presbyterian Hospital, Stribling et al. covered the Pediatric Consumer Librarian Service. This initiative involved librarians performing consumer rounds for the bedside delivery of health information, thus supporting PFCC initiatives through “information-sharing/patient education.” Anecdotal evidence supported that this program satisfied the PFCC outcomes of “improved patient/family experience” and “better clinician/staff satisfaction,” citing in-person, email, and survey-based feedback from both families and providers [11].

Donahue et al. likewise described a hospital-based intervention that supported the PFCC initiatives of “information-sharing/patient education” and “participatory care/decision-making.” Responding to the information needs of patients, librarians from the Aurora Health Care System created a new consumer health service wherein they visited hospital floors for patient information rounds. During these information rounds, librarians rounded on floors to offer custom-tailored information delivery that kept patients and families informed and empowered to make decisions about their care. Outcomes reported by the authors aligned with the PFCC outcome “improved patient/family experience.” Patient feedback in these cases was measured by a patient rounding log, in which a librarian recorded answers to questions on patients’ experiences [8].

Contributions of the Family Resource Center in the Bristol-Myers Squibb Children’s Hospital were outlined by Forsberg. As part of the hospital’s dedication to family-centered care, she described how the librarian helps families with literature searches, information evaluation, and health literacy needs. This, again, illustrated a case in which librarians supported the PFCC initiatives “information-sharing/patient education” and “participatory care/decision-making” by making their services directly available to patients, families, and caregivers. PFCC outcomes of incorporating a librarian into the Family Resource Center were defined as “better health outcomes,” “improved patient/family experience,” and “other (empowerment)”; however, details on measuring these outcomes were not discussed [10].

Beschnett et al. explored library involvement in patient education at Allina Health, a nonprofit health system based in Minneapolis, Minnesota. Librarians at Allina Library Services provided high-quality, reliable health information and patient education materials to patients and clinical staff via email, in-person, and the patient portal (MyChart) of the system’s electronic medical record. Identified PFCC initiatives included “education of health professionals,” “information-sharing/patient education,” and “participatory care/decision-making.” As a result of the library’s involvement in consumer health outreach, recorded PFCC outcomes included “improved patient/family experience” [5].

## DISCUSSION

Consistent with the values of PFCC, all included articles identified patient education or information-sharing as an integral component of their initiatives. Librarians provided these services through one-on-one consultations, information prescription programs, and the creation of targeted patient information handouts. Patient participation in shared decision-making was identified in most studies. As health care decision-making requires informed patients, it is not surprising that the included PFCC cases utilized interventions to empower their patients. Librarians were noted to contribute to shared decision-making through consulting directly with patients, improving the health literacy of patients by teaching them how to evaluate health information, and delivering information to both providers and patients with the goal of fostering collaborative communication.

The findings of this scoping review suggest that librarians who are interested in PFCC can leverage their daily activities to contribute information to the Medicare Attestation Worksheet to document meaningful use requirements, which provide for reimbursement of patient-specific education under the Medicare Access and Chip Reauthorization Act of 2015 [17]. Specifically, stage 2 of the Eligible Professional Meaningful Use Core Measures outlines the requirements of organizations to perform electronic health record (EHR) tasks to achieve coordination of care. Measure 13 lays out the objective to “Use clinically relevant information from Certified EHR Technology to identify patient-specific education resources and provide those resources to the patient” [18]. The measure is used to ensure that patients are connected to health education resources that are relevant to their own cases in order to support patient engagement and quality of care. It further supports the Medicare EHR Incentive Program, which informs the allocation of resources through the Centers for Medicare & Medicaid Services’s (CMS’s) Merit Based Incentive Payment System. Librarians should, thus, seek to record their patient-centered educational activities in their institutions’ EHR to document meaningful use measures to support funding and CMS quality programs [19].

The inclusion of three separate Planetree health care facilities—Mid-Columbia Medical Center, The Dalles, Oregon; Griffin Hospital, Derby, Connecticut; and Cooper University Hospital, Camden, New Jersey—among the final studies warrants further discussion. The Planetree model is a not-for-profit collaboration of health care organizations that facilitate person-centered care through targeted education and information programs [20]. As the Planetree notion of patient-as-partner is consistent with PFCC values, its resources and programs deserve further investigation by information professionals who are interested in providing and promoting PFCC.

When examining reported PFCC outcomes among included studies, “improved patient/family experience” was reported by all studies. The most robust method used by authors to gauge improved experience was patient and family survey data, coupled with informal feedback (noted in librarian or volunteer rounding logs). “Better health outcomes” and “better clinical/staff satisfaction” were noted in several studies; however, connections between PFCC initiatives and health outcomes or staff satisfaction should be evaluated cautiously, because evaluative data were often limited to surveys of small convenience samples or anecdotal evidence. As the adequacy and appropriateness of reported librarian-involved initiatives were not the result of rigorous study design or measure, it was difficult to accurately appraise the effect and actual impact of the interventions described.

A limitation of this scoping review was our inclusion of only English-language articles. The inclusion of an Italian article written in English highlighted the possibility of other international librarian-involved PFCC initiatives that might have been reported in other languages and were not captured by our search. An additional limitation of our scoping review concerned the terminology that we relied upon during the full-text review phase. Specifically, we included and extracted data only from articles that used the terms “patient and family centered care,” “patient centered care,” “family centered care,” “patient focused care,” or “family focused care,” including spelling variations. We are aware many articles were excluded because they described methods or programs closely related to PFCC but did not use the aforementioned terms. Outcome evaluation of the activities described in each article was often limited to anecdotal evidence. The adequacy or appropriateness of librarian-involved initiatives were not the result of rigorous study design or measure, as all included studies were case reports, which provide a low level of evidence. In particular, it was difficult to ascertain the PFCC outcome “better health outcomes” through the type of data presented in the included studies.

This scoping review is a starting point for librarians and information professionals to recognize ways of getting involved in PFCC programs in their hospitals and medical centers. The synthesis of available evidence to date suggests that librarians and information professionals should focus on patient education and information-sharing to support both patients or caregivers and clinical staff. The burgeoning efforts in participatory care and inclusion of patients in the decision-making process pose a unique opportunity for librarians and information professionals to offer more personalized information services. Librarians and information professionals should work in partnership with clinical and administrative staff to focus on developing information interventions to enhance the patient experience and improve outcomes, including measuring and evaluating outcomes.

Opportunities for further research in this area include investigating librarian or information professional involvement in PFCC from an international perspective. A global approach to understanding librarian- or information professional–led PFCC programs can help to offer more culturally competent and less language-dependent information services to patients or caregivers. The literature would also benefit from more data-driven studies in the arena of PFCC from the librarian or information professional’s point of view. The studies included in this scoping review lack rigorous methodologies and data-centric assessments. The anecdotal evidence for PFCC programs and information services can be expanded by use of more thorough methodological techniques to better link the interventions and involvement to specified outcomes.

## SUPPLEMENTAL FILES

Appendix A Complete list of Medical Subject Headings (MeSH) terms and keywords used for the MEDLINE search strategy

Appendix B Code sheet for librarians supporting patient- and family-centered care (PFCC)

Appendix C Details of the twelve studies that met the criteria for inclusion

## References

[1] Fix GM, VanDeusen Lukas C, Bolton RE, Hill JN, Mueller N, LaVela SL, Bokhour BG (2018). Patient-centred care is a way of doing things: how healthcare employees conceptualize patient-centred care. Health Expect.

[2] Johnson B, Abraham M (2012). Partnering with patients, residents, and families: a resource for leaders of hospitals, ambulatory care settings, and long-term care communities.

[3] Anglin C (2008). Providing pediatric psychosocial support through patient library services in an outpatient hematology/oncology clinic. Prim Psychiatry.

[4] Babish JA (2002). CHI services: Planetree—a patient-centered care concept. Natl Netw (Dallas, TX).

[5] Beschnett A, Bulger J (2013). Patient education and the hospital library: opportunities for involvement. J Hosp Librariansh.

[6] Calabretta N, Cavanaugh S, Malone M, Swartz BJ (2011). A hospital-based patient and family education center: if you build it, will they come?. Med Ref Serv Q.

[7] Davis J (2013). Health information ambassador program for patient education: a best practice for bringing the consumer health library to the patient. J Consumer Health Internet.

[8] Donahue A, Budzisz V, Egebo H, Fay B, Karnold S, Koepsel M, Lisiecki MA, Prasad M, Ruby D, Strube K, Webb L (2012). Consumer health outreach as a sum of parts: individual and collective approaches of a health care system’s libraries. J Hosp Librariansh.

[9] Editors (1997). Hospital’s education center encourages self-care in style. Healthc Demand Dis Manag.

[10] Forsberg NN (2010). Family friendly space for research, reflection, and respite: a family resource center and library in a pediatric hospital setting. J Hosp Librariansh.

[11] Stribling JC, Mages KC, Delgado D (2017). Patient-centered rounding in an inpatient pediatric setting. J Hosp Librariansh.

[12] Tarby W, Hogan K (1997). Hospital-based patient information services: a model for collaboration. Bull Med Libr Assoc.

[13] Truccolo I (2016). Providing patient information and education in practice: the role of the health librarian. Health Inf Libr J.

[14] Williams MD, Gish KW, Guise NB, Sathe NA, Carrell DL (2001). The Patient Informatics Consult Service (PICS): an approach for patient-centered service. Bull Med Libr Assoc.

[15] Moher D, Liberati A, Tetzlaff J, Altman DG (2009). Preferred reporting items for systematic reviews and meta-analyses: the PRISMA statement. PLoS Med.

[16] McKnight M (2014). Information prescriptions, 1930–2013: an international history and comprehensive review. J Med Libr Assoc.

[17] Rathert C, Porter TH, Mittler JN, Fleig-Palmer M (2019). Seven years after meaningful use: physicians’ and nurses’ experiences with electronic health records. Health Care Manage Rev.

[18] Centers for Medicare & Medicaid Services (2012). Eligible professional: meaningful use core measures.

[19] Shipman JP, Lake EW, Van Der Volgen J, Doman D (2016). Provider documentation of patient education: a lean investigation. J Med Libr Assoc.

