# Supplementary material for: Involvement of information professionals in patient- and family-centered care initiatives: a scoping review
Source: J Med Libr Assoc. 2019 Jul 1;107(3):314–22. doi: 10.5195/jmla.2019.652 (PMC6579588; doi:10.5195/jmla.2019.652)
Supplement: Appendix A [file jmla-107-314-s001.pdf]

JMLA Journal of the Medical Library Association

Supplemental content to
J Med Libr Assoc. Jul;107(3):dx.doi.org/10.5195/jmla.2019.652
www.jmla.mlanet.org
© DeRosa, Nelson, Delgado, Mages, Baltich Martin, Stribling 2019

Involvement of information professionals in patient- and family-centered care initiatives: a scoping review

Antonio P. DeRosa, MS, MLIS, AHIP; Becky Baltich Nelson, MS, MLS; Diana Delgado, MLS, AHIP; Keith C. Mages, PhD, MLS, MSN, RN, AHIP; Lily Martin, MLS; Judy C. Stribling, MS, MLS, AHIP

APPENDIX A

Complete list of Medical Subject Headings (MeSH) terms and keywords used for the MEDLINE search strategy

1. exp LIBRARIES/
2. library.tw.
3. libraries.tw.
4. LIBRARIANS/
5. librarian*.tw.
6. informationist*.tw.
7. information professional.tw.
8. information professionals.tw.
9. information specialist.tw.
10. information specialists.tw.
11. information scientist.tw.
12. information scientists.tw.
13. exp Library Services/
14. interlibrary loan*.tw.
15. Library Science/
16. Information Centers/
17. information center.tw.
18. information centers.tw.
19. information centre.tw.
20. information centres.tw.
21. Information Services/
22. information service.tw.
23. information services.tw.
24. online service.tw.
25. online services.tw.
26. or/1-25
27. exp Patient-Centered Care/
28. patient centered.tw.
29. patient centred.tw.
30. patient focused.tw.
31. family centered.tw.

JMLA Journal of the Medical Library Association

SC
Supplemental content to
J Med Libr Assoc. Jul;107(3):dx.doi.org/10.5195/jmla.2019.652
www.jmla.mlanet.org
© DeRosa, Nelson, Delgado, Mages, Baltich Martin, Stribling 2019

32. family centred.tw.
33. PCC.tw.
34. PFCC.tw.
35. patient navigation*.tw.
36. patient navigator*.tw.
37. narrative medicine.tw.
38. patient care.tw.
39. continuity of care.tw.
40. episode of care.tw.
41. patient helper*.tw.
42. patient management.tw.
43. Patient Compliance/
44. patient compliance.tw.
45. patient cooperation.tw.
46. patient noncompliance.tw.
47. patient non-compliance.tw.
48. patient nonadherence.tw.
49. patient non-adherence.tw.
50. therapeutic compliance.tw.
51. treatment compliance.tw.
52. adherence to therapy*.tw.
53. adherence to treatment.tw.
54. compliance to therapy.tw.
55. compliance to treatment.tw.
56. patient's adherence.tw.
57. therapy adherence.tw.
58. treatment adherence.tw.
59. medication compliance.tw.
60. Patient Participation/
61. patient participation.tw.
62. patient involvement.tw.
63. patient empowerment.tw.
64. patient activation.tw.
65. patient engagement.tw.
66. consumer participation.tw.
67. patient decision making.tw.
68. shared decision making.tw.
69. advance care planning.tw.
70. Professional-Patient Relations/

SC
Supplemental content to
J Med Libr Assoc. Jul;107(3):dx.doi.org/10.5195/jmla.2019.652
www.jmla.mlanet.org
© DeRosa, Nelson, Delgado, Mages, Baltich Martin, Stribling 2019

71. professional patient relation*.tw.
72. contacting client*.tw.
73. recontact*.tw.
74. duty to follow up.tw.
75. Patient Education as Topic/
76. patient education.tw.
77. education of patient*.tw.
78. Patient Advocacy/
79. patient advocacy.tw.
80. patient ombudsman.tw.
81. patient ombudsmen.tw.
82. patient representative*.tw.
83. clinical ombudsman.tw.
84. clinical ombudsmen.tw.
85. Models, Organizational/
86. organizational model*.tw.
87. Cooperative Behavior/
88. cooperative behavior*.tw.
89. cooperative behaviour*.tw.
90. compliant behavior*.tw.
91. compliant behaviour*.tw.
92. helping behavior*.tw.
93. helping behaviour*.tw.
94. or/27-93
95. 26 and 94
