# Supplementary material for: Involvement of information professionals in patient- and family-centered care initiatives: a scoping review
Source: J Med Libr Assoc. 2019 Jul 1;107(3):314–22. doi: 10.5195/jmla.2019.652 (PMC6579588; doi:10.5195/jmla.2019.652)
Supplement: Appendix B [file jmla-107-314-s002.pdf]

JMLA Journal of the Medical Library Association

Supplemental content to
J Med Libr Assoc. Jul;107(3):dx.doi.org/10.5195/jmla.2019.652
www.jmla.mlanet.org
© DeRosa, Nelson, Delgado, Mages, Baltich Martin, Stribling 2019

Involvement of information professionals in patient- and family-centered care initiatives: a scoping review

Antonio P. DeRosa, MS, MLIS, AHIP; Becky Baltich Nelson, MS, MLS; Diana Delgado, MLS, AHIP; Keith C. Mages, PhD, MLS, MSN, RN, AHIP; Lily Martin, MLS; Judy C. Stribling, MS, MLS, AHIP

APPENDIX B

Code sheet for librarians supporting patient- and family-centered care (PFCC)

**Paper characteristics**		
P1. Study ID number	#	____
P2. Coder	BLINDED FOR PEER REVIEW	____
P3. Author(s)	*Last, First et al.*	
P4. Date of publication	Year	____
P5. Location of study	0=USA	____
	1=Other country________________	____
**Study design**		
S1. Study design	0=Systematic review	____
	1=Meta-analysis	
	2=Review	
	3=Case report	
	4=Clinical trial	
	5=Randomized controlled trial (RCT)	
	6=Observational study	
	7=Exploratory/field study	
	8=Conference abstract	
	9=Other ______________________	

JMLA Journal of the Medical Library Association

SC
Supplemental content to
J Med Libr Assoc. Jul;107(3):dx.doi.org/10.5195/jmla.2019.652
www.jmla.mlanet.org
© DeRosa, Nelson, Delgado, Mages, Baltich Martin, Stribling 2019

S2. Analysis methods	0=Quantitative<br>1=Qualitative<br>2=Mixed-methods<br>3=No analysis (e.g., review articles)	____
**Study characteristics**		
C1. Population	0=Patients<br>1=Family/caregivers<br>2=Public/community<br>3=Mixed<br>4=Other ________________________	____
C2. Type of patient- and family-centered care (PFCC) initiative (from the Institute of PFCC definition of PFCC: www.ipfcc.org/about/pfcc.html)	0=Quality/service improvement<br>1=Safety initiatives<br>2=Education of health professionals<br>3=Research<br>4=Facility design<br>5=Policy development<br>6=Information sharing/patient education<br>7=Participatory care/decision-making<br>8=Cultural and spiritual competencies<br>9=Other ________________________	____
C3. If C2=*9* (*other)*	Describe program/project discussed in study (we will synthesize into concepts/themes after data extraction phase)	

JMLA Journal of the Medical Library Association

SC
Supplemental content to
J Med Libr Assoc. Jul;107(3):dx.doi.org/10.5195/jmla.2019.652
www.jmla.mlanet.org
© DeRosa, Nelson, Delgado, Mages, Baltich Martin, Stribling 2019

C4. Type of institution (where PFCC study took place)	0=Hospital<br>1=University/college<br>2=Nonprofit organization<br>3=Public library<br>4=Federal institution<br>5=Company/business<br>6=Other ________________	____
C5. Information professional role/intervention	Describe the role/intervention that the information professional played in supporting the PFCC program or project (we will synthesize into concepts/themes after data extraction phase)	
C6. How information professional or library became involved	Describe how the information professional or library became involved in supporting the PFCC program or project (we will synthesize into concepts/themes after data extraction phase)	
C7. Library as place or librarian as professional?	0=Library or information/resource center<br>1=Librarian or information professional (various titles)<br>2=Both library and librarian<br>3=Other ______________________	____
C8. PFCC outcomes (from the Institute of PFCC definition of PFCC: www.ipfcc.org/about/pfcc.html)	0=Better health outcomes<br>1=Improved patient/family experience<br>2=Better clinician/staff satisfaction<br>3=Wiser allocation of resources<br>4=Other ______________________	____
C9. If C8=4 *(other)*	Describe any/all outcomes discussed in the study that do not appear in C8; e.g., empowerment, health literacy, reduced cost/time, other collaborative and participatory outcomes, etc. (we will synthesize into concepts/themes after data extraction phase)	
