# Supplementary material for: Involvement of information professionals in patient- and family-centered care initiatives: a scoping review
Source: J Med Libr Assoc. 2019 Jul 1;107(3):314–22. doi: 10.5195/jmla.2019.652 (PMC6579588; doi:10.5195/jmla.2019.652)
Supplement: Appendix C [file jmla-107-314-s003.pdf]

JMLA Journal of the Medical Library Association

Supplemental content to
J Med Libr Assoc. Jul;107(3):dx.doi.org/10.5195/jmla.2019.652
www.jmla.mlanet.org
© DeRosa, Nelson, Delgado, Mages, Baltich Martin, Stribling 2019

Involvement of information professionals in patient- and family-centered care initiatives: a scoping review

Antonio P. DeRosa, MS, MLIS, AHIP; Becky Baltich Nelson, MS, MLS; Diana Delgado, MLS, AHIP; Keith C. Mages, PhD, MLS, MSN, RN, AHIP; Lily Martin, MLS; Judy C. Stribling, MS, MLS, AHIP

APPENDIX C

Details of the twelve studies that met the criteria for inclusion

Author	Year	Study design	Population	Patient- and family-centered care (PFCC) category*	Institution type	Information professional interventions	How information professional became involved	PFCC outcomes*
Anglin C	2008	Case report	Mixed	• Safety initiatives<br>• Education of health professionals<br>• Facility design<br>• Policy development<br>• Information sharing/patient education<br>• Participatory care/decision-making<br>• Cultural and spiritual competencies	University/ college	Librarians address issues to assist patients in making "evidence-based patient choices." Areas include health literacy, school readiness, psychosocial issue support, research support for patients and clinicians.	Invited to be part of the integrated care team	• Better health outcomes<br>• Improved patient/ family experience<br>• Better clinician/ staff satisfaction

Supplemental content to
J Med Libr Assoc. Jul;107(3):dx.doi.org/10.5195/jmla.2019.652
www.jmla.mlanet.org
© DeRosa, Nelson, Delgado, Mages, Baltich Martin, Stribling 2019

Author	Year	Study design	Population	Patient- and family-centered care (PFCC) category*	Institution type	Information professional interventions	How information professional became involved	PFCC outcomes*
Babish J	2002	Case report	Mixed	• Information sharing/patient education<br>• Participatory care/decision-making	Hospital	Follows the Planetree Model for empowering patients through information and education. Involved in health literacy outreach programming and cultural competency services.	Librarian-initiated programming in accordance with the Planetree Model mandate to provide consumer health information and education.	• Improved patient/ family experience
Beschnett A	2013	Case report	Mixed	• Education of health professionals<br>• Information sharing/patient education<br>• Participatory care/decision-making	Hospital	Provide quality and reliable health information and patient education materials to patients as well as clinical staff via the library electronically, in-person, and the patient portal (MyChart) of the electronic medical record (EMR).	Participation in Patient Education Council	• Improved patient/ family experience
Calabretta N et al.	2011	Case report	Mixed	• Education of health professionals<br>• Information sharing/patient education	Hospital	At the Patient and Family Education Center (PFEC), librarians provide patient-centered health information to patients, families/caregivers, and staff to ease and enrich the patient experience. Librarians rotate through the PFEC in 4-hour shifts to be available to patients, families, and caregivers.	As a Planetree affiliate, the hospital's senior administration approached library director with the opportunity to plan and manage the PFEC space.	• Improved patient/ family experience<br>• Better clinician/ staff satisfaction<br>• Wiser allocation of resources

JMLA Journal of the Medical Library Association

SC
Supplemental content to
J Med Libr Assoc. Jul;107(3):dx.doi.org/10.5195/jmla.2019.652
www.jmla.mlanet.org
© DeRosa, Nelson, Delgado, Mages, Baltich Martin, Stribling 2019

Author	Year	Study design	Population	Patient- and family-centered care (PFCC) category*	Institution type	Information professional interventions	How information professional became involved	PFCC outcomes*
Davis J	2013	Case report	Mixed	• Information sharing/patient education<br>• Participatory care/decision-making	Hospital	Volunteers take questions from patients as part of the Health Information Ambassador Program and bring them back to librarians who answer specific requests and follow up with patients. Packets are put together and delivered to patients in-person and electronically.	Librarian-initiated program. Ambassadorship program created as a result of work done on the hospital's Patient & Family Centered Care Team.	• Improved patient/ family experience<br>• Empowerment†
Donahue A et al.	2012	Case report	Mixed	• Information sharing/patient education<br>• Participatory care/decision-making	Hospital	Delivery of patient education materials to patients and families. Librarians round on floors offering custom-tailored information delivery; keeping patients and families informed and empowered to make decisions about care.	Librarian-initiated program. Effort by whole Aurora Health Care library system to offer consumer health information services to patient, families/ caregivers, and the community.	• Improved patient/ family experience

Supplemental content to
J Med Libr Assoc. Jul;107(3):dx.doi.org/10.5195/jmla.2019.652
www.jmla.mlanet.org
© DeRosa, Nelson, Delgado, Mages, Baltich Martin, Stribling 2019

Author	Year	Study design	Population	Patient- and family-centered care (PFCC) category*	Institution type	Information professional interventions	How information professional became involved	PFCC outcomes*
Editors of *Healthcare Demand & Disease Management*	1997	Case report	Mixed	• Education of health professionals<br>• Information sharing/patient education	Hospital	Librarians provide health information materials to patients, public, and health professionals and oversee the coordination of educational lectures for the public/patient populations.	Health Resource Center is an outgrowth of Planetree Health System, a California-based initiative that is modeled on patient-focused care, prevention, education, and empowerment.	• Improved patient/ family experience
Forsberg NN et al.	2010	Case report	Mixed	• Information sharing/patient education<br>• Participatory care/decision-making	Hospital	Family resource librarian works at the Family Resource Center in the Bristol-Myers Squibb Children's Hospital, helping families with literature searches, information evaluation, and health literacy needs.	The librarian was incorporated into the Family Resource Center and library to improve family-centered care (unclear whether or not this position was present at the center's opening or was created later).	• Better health outcomes<br>• Improved patient/ family experience<br>• Empowerment†

JMLA Journal of the Medical Library Association

SC
Supplemental content to
J Med Libr Assoc. Jul;107(3):dx.doi.org/10.5195/jmla.2019.652
www.jmla.mlanet.org
© DeRosa, Nelson, Delgado, Mages, Baltich Martin, Stribling 2019

Author	Year	Study design	Population	Patient- and family-centered care (PFCC) category*	Institution type	Information professional interventions	How information professional became involved	PFCC outcomes*
Stribling JC et al.	2017	Case report	Mixed	• Information sharing/patient education	Hospital; university/ college	Librarian performs consumer rounds for bedside delivery of health information	Librarian-initiated program. Recognized the need to resume/ improve consumer support for pediatrics after a conversation between the library's assistant director and the Department of Pediatrics chair of quality and patient safety.	• Improved patient/ family experience<br>• Better clinician/ staff satisfaction
Tarby W et al.	1997	Case report	Mixed	• Education of health professionals<br>• Information sharing/patient education<br>• Participatory care/decision-making	Hospital	Collaborative work with patient education coordinators to devise a way for patients and clinicians to request consumer health information. Patients are able to make information requests directly with librarians and able to meet with them to pursue specific information needs.	Patient Education Task Force was created and librarians invited to participate. Librarians conduct information needs assessment of patients and clinicians.	• Better health outcomes<br>• Improved patient/ family experience

JMLA Journal of the Medical Library Association

Supplemental content to
J Med Libr Assoc. Jul;107(3):dx.doi.org/10.5195/jmla.2019.652
www.jmla.mlanet.org
© DeRosa, Nelson, Delgado, Mages, Baltich Martin, Stribling 2019

Author	Year	Study design	Population	Patient- and family-centered care (PFCC) category*	Institution type	Information professional interventions	How information professional became involved	PFCC outcomes*
Truccolo I	2016	Case report	Mixed	• Education of health professionals<br>• Information sharing/patient education	Hospital	Librarian provides patient education handouts to patients and families/caregivers with the aim of improving the dialogue between patients and providers. Librarian is also the coordinator of a narrative medicine program, which seeks to give a voice to patients and teaches experts to listen carefully and use plain language when communicating health information.	Scientific director of the institution established a Patient Education & Empowerment Group (PEEG), charging the health librarian with the role of technical coordinator of the team and overall program.	• Improved patient/ family experience

JMLA Journal of the Medical Library Association

SC
Supplemental content to
J Med Libr Assoc. Jul;107(3):dx.doi.org/10.5195/jmla.2019.652
www.jmla.mlanet.org
© DeRosa, Nelson, Delgado, Mages, Baltich Martin, Stribling 2019

Author	Year	Study design	Population	Patient- and family-centered care (PFCC) category*	Institution type	Information professional interventions	How information professional became involved	PFCC outcomes*
Williams MD et al.	2001	Case report	Mixed	• Information sharing/patient education<br>• Participatory care/decision-making	Hospital; university/ college	Patient Informatics Consult Service (PICS) librarians developed an information prescription form to be filled out by physicians. In response to an information prescription, they appraise and summarize consumer information into a single and personalized report (includes different viewpoints, appropriate reading level, highlight relevant sections, provide brief summary of each item, etc.). The report is given to both physician and patient to ease doctor-patient communication and create a dialogue. PICS librarians also create web-based pathfinders on prescription topics (which include disease overviews, links to authoritative resources, etc.)	Librarian-initiated program. The information prescription program drew on success of an earlier Clinical Informatics Consult Service (CICS), which placed librarians with clinical teams on rounds.	• Better health outcomes<br>• Improved patient/ family experience<br>• Better clinician/ staff satisfaction<br>• Empowerment†

* PFCC categories and outcomes according to the Institute for Patient- and Family-Centered Care (IPFCC) definition of patient-centered care.
† *Other* PFCC category or outcomes described in study that are not included in the IPFCC definition.
